# Distinctive Role of the Systemic Inflammatory Profile in Non-Small-Cell Lung Cancer Younger and Elderly Patients Treated with a PD-1 Immune Checkpoint Blockade: A Real-World Retrospective Multi-Institutional Analysis

**DOI:** 10.3390/life11111235

**Published:** 2021-11-15

**Authors:** Valerio Nardone, Rocco Giannicola, Diana Giannarelli, Rita Emilena Saladino, Domenico Azzarello, Caterina Romeo, Giovanna Bianco, Maria Rosaria Rizzo, Irene Di Meo, Antonio Nesci, Pierpaolo Pastina, Antonia Consuelo Falzea, Daniele Caracciolo, Alfonso Reginelli, Michele Caraglia, Amalia Luce, Luciano Mutti, Antonio Giordano, Salvatore Cappabianca, Luigi Pirtoli, Vito Barbieri, Pierfrancesco Tassone, Pierosandro Tagliaferri, Pierpaolo Correale

**Affiliations:** 1 Department of Precision Medicine, University of Campania “L. Vanvitelli”, 80138 Naples, Italy; alfonso.reginelli@unicampania.it (A.R.); michele.caraglia@unicampania.it (M.C.); amalia.luce@unicampania.it (A.L.); salvatore.cappabianca@unicampania.it (S.C.); 2 Medical Oncology Unit, Grand Metropolitan Hospital “Bianchi-Melacrino-Morelli”, 89124 Reggio Calabria, Italy; roccogiannicola@gmail.com (R.G.); domenico.azzarello@libero.it (D.A.); caterina.romeo@libero.it (C.R.); giovannabianco@libero.it (G.B.); antonellafalzea@gmail.com (A.C.F.); correalep@yahoo.it (P.C.); 3 Biostatistical Unit, National Cancer Institute “Regina Elena”, IRCCS, 00161 Rome, Italy; diana.giannarelli@ifo.gov.it; 4 Tissue typing Unit, Grand Metropolitan Hospital “Bianchi-Melacrino-Morelli”, 89124 Reggio Calabria, Italy; ritaemilena.saladino@gmail.com; 5 Department of Advanced Medical and Surgical Sciences, University of Campania “Luigi Vanvitelli”, 80138 Naples, Italy; mariarosaria.rizzo@unicampania.it (M.R.R.); irenedimeo@libero.it (I.D.M.); 6 Unit of Pharmacy, Grand Metropolitan Hospital “Bianchi-Melacrino-Morelli”, 89124 Reggio Calabria, Italy; antonio.nesci@gmail.com; 7 Section of Radiation Oncology, Medical School, University of Siena, 53100 Siena, Italy; pastina.pierpaolo85@gmail.com; 8 Medical and Translational Oncology Unit, Department of Experimental and Clinical Medicine, Magna Graecia University, 88100 Catanzaro, Italy; daniele.caracciolo@unicz.it (D.C.); v.barbieri@materdominiaou.it (V.B.); tassone@unicz.it (P.T.); tagliaferri@unicz.it (P.T.); 9 BiogemScarl, Institute of Genetic Research, Precision and Molecular Oncology Laboratory, Ariano Irpino, 83031 Avellino, Italy; 10 Sbarro Institute for Cancer Research and Molecular Medicine and Center of Biotechnology, College of Science and Technology, Temple University, Philadelphia, PA 19122, USA; luciano.mutti@hotmail.it (L.M.); giordano@temple.edu (A.G.); luigipirtoli@gmail.com (L.P.); 11 Department of Medical Biotechnology, University of Siena, 53100 Siena, Italy

**Keywords:** immune checkpoint blockade, metastatic non-small-cell lung cancer, real-world evidence study, age, inflammatory markers, immunotherapy, immunosenescence

## Abstract

An immune checkpoint blockade with mAbs to PD-1 and PD-L1 is an expanding therapeutic option for mNSCLC patients. This treatment strategy is based on the use of mAbs able to restore the anti-tumor activity of intratumoral T cells inhibited by PD-1 binding to PD-L1/2 on tumor and inflammatory cells. It has been speculated that a chronic status of systemic inflammation as well as the immunosenescence physiologically occurring in elderly patients may affect the efficacy of the treatment and the occurrence of irAEs. We performed a multi-institutional retrospective study aimed at evaluating the effects of these mAbs (nivolumab or atezolizumab) in 117 mNSCLC patients younger (90 cases) and older (27 cases) than 75 years in correlation with multiple inflammatory parameters (NLR, CRP, ESR, LDH and PCT). No differences were observed when the cohorts were compared in terms of the frequency of PFS, OS, inflammatory markers and immune-related adverse events (irAEs). Similarly, the occurrence of irAEs was strictly correlated with a prolonged OS survival in both groups. On the contrary, a negative correlation between the high baseline levels of inflammatory markers and OS could be demonstrated in the younger cohort only. Overall, PD-1/PD-L1-blocking mAbs were equally effective in young and elderly mNSCLC patients; however, the detrimental influence of a systemic inflammation at the baseline was only observed in young patients, suggesting different aging-related inflammation immunoregulative effects.

## 1. Introduction

A peripheral immune checkpoint blockade (ICB) with mAbs to the programmed cell death receptor-1 (PD-1; nivolumab or pembrolizumab) and to its main ligand (PD-L1; atezolizumab, durvalumab or avelumab) alone or in combination with a number of different chemo-radiation strategies is an expanding therapeutic option for common malignancies including metastatic non-small-cell lung cancer (mNSCLC), malignant melanomas, head and neck carcinomas and urothelial and kidney cancer [1,2].

These immune-based treatments may be very effective but they are also associated with frequent immune-related adverse events (irAEs) and high costs [3,4,5,6,7]. 

Although the efficacy of these treatments in mNSCLC patients has been demonstrated in several randomized clinical trials, poor information is available for the population of patients older than 75 years who may have frequent comorbidities and a compromised T cell-mediated immune response due to the physiological immunosenescence process. 

In this light, there is a large amount of evidence showing a physiologically-based immunological impairment progressively occurring along with aging. This phenomenon, termed immunosenescence, has become an active field of investigation with the expanding use of an ICB in the treatment of human malignancies. It consists of a poorly characterized progressive decline of all immune functions related to a dynamic process of immune remodeling and adaptation naturally occurring along with the whole lifespan of each individual patient [8,9]. In this context, several pathogens such as the respiratory syncytial virus, influenza and para-influential viruses or Sars-Cov-2 that generally present a milder infection course in younger individuals may become fatal in elderly patients [10,11,12]. 

In light of this knowledge, many clinical investigators have hypothesized that both the efficacy and the safety of PD-1/PD-L1-blocking mAbs might significantly change in mNSCLC patients in parallel with their own aging. A large amount of data may be cautiously collected from those patients who have received these treatments in a real-world setting [12], showing good results in comparison with younger patients in terms of both OS and the safety profile [13].

We performed, therefore, a retrospective multi-institutional age-related analysis of mNSCLC patients who underwent a PD-1/PD-L1 mAbs treatment and correlated their outcome with multiple clinical, biological and inflammatory markers.

## 2. Materials and Methods

### 2.1. Patients, Treatment and Monitoring

This work was part of a retrospective real-world evidence (RWE) multi-institutional database study that included 117 chemo-refractory mNSCLC patients consecutively enrolled to receive a salvage therapy with an anti-PD-1 (nivolumab) or an anti-PD-L1 (atezolizumab) mAb at the OU-RC, MOU-CZ and ROU-SI between September 2015 and April 2020 with a median follow-up time of 27 months [7,14]. 

The inclusion criterion was patients with mNSCLC with an age > 18 years who progressed during or after 1st line chemotherapy (platin doublets). 

The exclusion criteria were patients with a background of autoimmune diseases in an active phase (such as ulcerative colitis and Crohn’s disease with the exclusion of diabetes and thyroiditis) and patients who had a solid organ transplantation.

In our retrospective analysis, our patients were categorized into two cohorts including younger patients (90 cases) and patients older than 75 years (27 cases). For the whole population, the median age was 69 years, mean 67 ± 9 years, range 44–85 years. 

All patients gave an informed consent for the anonymous use of their clinical data for the research aim. All procedures were undertaken in compliance with the ethical statements of the Helsinki Declaration (1964, amended most recently in 2008) of the World Medical Association and respect of their privacy.

All patients received a PD-1/PD-L1 blockade in a real-world setting as recommended by the international guidelines and regulative agencies following the standard procedures of administration for each drug. All patients, according to their specific disease, received nivolumab (intravenous infusion of 3 mg/kg every two weeks) (84 patients) or atezolizumab (intravenous infusion of 1200 mg every three weeks) (33 patients) until the disease progression or the occurrence of severe adverse events. All patients were fit for treatment (Eastern Cooperative Oncology Group (ECOG) performance status ≤ 1). A complete physical examination report and histological sampling as well as hematologic, biochemical, immune-biological, radiological and instrumental monitoring were available at the baseline. The clinical history, physical examinations and records of adverse events were evaluated prior to each drug infusion. A CT scan was performed every 3 months or in any case of suspected progressive disease (PD) and evaluated according to the immune Response Evaluation Criteria in Solid Tumors (iRECIST 1.1) [15]. 

All patients were monitored for blood cell counts and biochemistry prior to each treatment course and were also monitored for their adrenal hormone profile, ACTH, TSH, thyroid hormones, anti-thyroid autoantibodies (AAbs), extractable nuclear antigen antibodies (ENA), anti-nucleus antibodies (ANA), anti-smooth cell antibodies (ASMA) and c/p-anti-neutrophil cytoplasmic antibodies (ANCA) each month from the beginning of the treatment as reported in a previous study [16,17,18].

### 2.2. Statistical Analysis

Both the baseline and ∆Parameters were compared in the two cohorts of patients with a chi-squared analysis. 

In order to perform a statistical correlation among the continuous inflammatory parameters at the baseline and the outcomes, we used the median values as a cut-off. For ∆Parameters, the cut-off was set as 0, dividing the cohort into two subsets of patients who showed an increase or a decrease in the parameters during the immunotherapy. The time to events were analyzed with the Kaplan–Meier method and the statistics were performed by a log-rank test. The median survival and 95% confidence intervals were reported. The median follow-up was estimated with the reverse method. Hazard ratios (HRs) and their 95% confidence intervals were estimated through the Cox regression proportional model; in the multi-variate approach, a forward stepwise procedure was used and the enter and remove limits were set to 0.05 and 0.10, respectively.

The association of the frequency of irAEs with the biological parameters and the clinical outcome in the two patient cohorts was assessed by a chi-squared test. The statistics were performed by SPSS software 23.0 (International Business Machines Corp., New York, NY, USA).

## 3. Results

### 3.1. Features and Clinical Outcomes of the Patients

**Figure 1:**
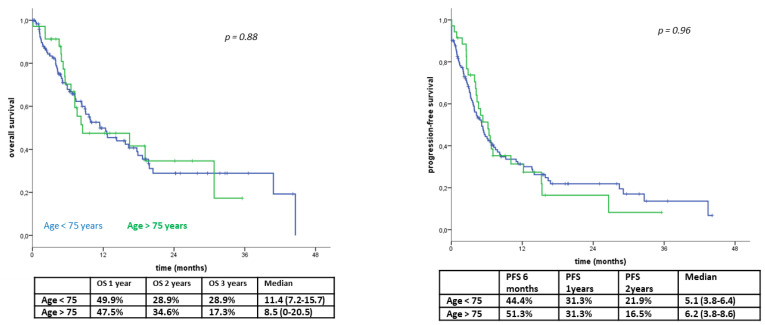
Kaplan–Meyer curves. (**a**) Overall survival (OS) and (**b**) progression-free survival (PFS) of metastatic non-small-cell lung cancer (mNSCLC) patients under treatment with nivolumab or atezolizumab divided into two age-related groups: age < 75 years (blue) and age > 75 years (green). In our series, we did not find any correlation with age for both overall survival (OS, patients ≤ 74 years median OS 11.4 months, 95% CI 7.2–15.7 months vs. patients ≤ 74 years median OS 8.5 months, 95% CI 0–20.5 months, *p*-value: 0.88) and progression-free survival (PFS, patients ≤ 74 years median PFS 5.1 months, 95% CI 3.8–6.4 months vs. patients ≥ 75 years median OS 6.2 months, 95% CI 3.8–8.6 months, *p*-value: 0.88).

Our retrospective analysis was performed on a cohort of 117 patients with mNSCLC who had been consecutively enrolled to receive a salvage therapy with nivolumab or atezolizumab between November 2015 and April 2020. In our series, there were 97 males and 20 females and 27 cases older than 75 years (Table 1). We recorded a median PFS and OS of 9.2 (95% CI; 5.4–13.0) and 17.9 (95% CI; 15.0–20.8) months, respectively, with a median follow-up of 27 months. We compared both clinical parameters among the two age-related cohorts of patients and found no difference in term of PFS (≤ 74 vs. ≥ 75-year-old = 8.3 (4.1–12.5) vs. 10.1 (2.0–18.2), *p* = 0.63) and OS (≤ 74 vs. ≥ 75-year-old = 17.9 (14.9–20.9) vs. 18.2 (8.8–27.6), *p* = 0.90) (Figure 1a,b).

Additionally, no differences in terms of PFS (*p*: 0.56) and OS (*p*: 0.42) were seen in the patients receiving anti-PD-1 vs. the patients receiving anti-PD-L1. 

#### 3.1.1. Baseline Systemic Inflammation Status and the Occurrence of Autoimmunity 

We subsequently evaluated the baseline inflammatory status in the two cohorts and found no difference in the baseline values of NLR, CRP, ESR, LDH and procalcitonin (PCT) as well as of treatment-related changes in the neutrophil counts (baseline vs. third treatment cycle—Delta N) (Table 1). This selection of inflammation biomarkers was based on the screening protocol that was used before each treatment course [6,16,17,19] although other inflammation biomarkers deserved to be investigated (serum amyloid A, cytokines, alpha-1-acid glycoprotein, plasma viscosity, ceruloplasmin and hepcidin among others). We compared the frequency of irAEs between the two cohorts of patients. In our series, the majority of adverse events were g 1–2, mainly involving poly-arthropathy in 70% of all irAE cases, thyroiditis in 25% of all irAE cases, autoimmune pneumonitis in 15% of the cases and reversible deficiency of the adrenal gland in 10% of the cases. There was contemporary evidence of multiple irAEs and no cases of hypophysis, ulcerative colitis or Crohn’s disease were recorded. We found no significant difference in the risk of autoimmunity (38.9% (35/90 cases) vs. 37.0% (10/27 cases), *p* = 0.86). 

#### 3.1.2. Prognostic Influence of the Baseline Systemic Inflammation Status and the Occurrence of Autoimmunity in Patients Treated with PD-1-Blocking mAbs

**Figure 2:**
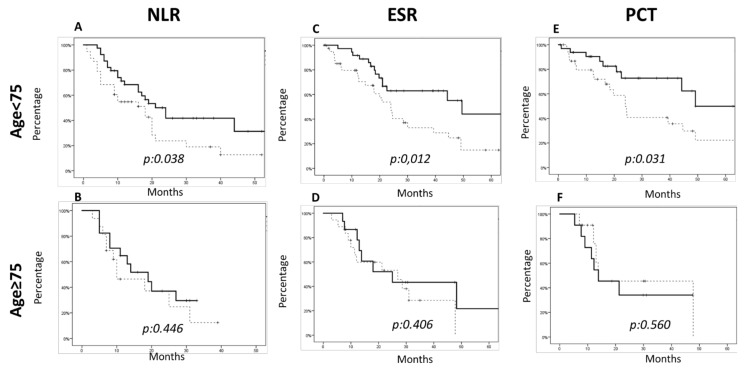
Overall survival considering the inflammation status parameters among the two cohorts of patients. The analysis showed that in the younger patient cohort (age ≤ 74 years), the parameters that were statistically significant were: (**A**,**B**) the neutrophile to lymphocyte ratio (NLR) (NLR < median value median OS of 24 months vs. NLR > median value median OS of 18 months, *p*-value 0.038); (**C**,**D**) erythrocyte sedimentation rate (ESR) (ESR < median value median OS of 49.5 months vs. ESR > median value of 24 months, *p*-value 0.012); and (**E**,**F**) procalcitonin (PCT) (PCT < median value of 49 months vs. PCT > median value of 24 months, *p*-value 0.031). In the older patient cohort (age ≥ 75 years), these parameters were not significantly correlated.

**Figure 3:**
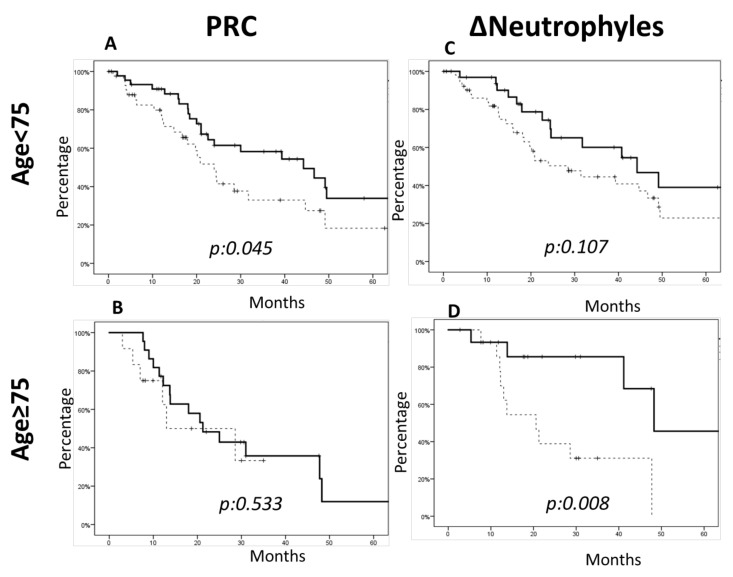
Overall survival considering the inflammation status parameters among the two cohorts of patients. The analysis showed that in the younger patient cohort (age ≤ 74 years), the C-reactive protein (CRP) parameter (**A**,**B**) was statistically significant (CRP < median value of 44 months vs. CRP > median value of 24 months, *p*-value 0.045). In the older patient cohort (age ≥ 75 years), the only parameter that significantly correlated with a greater survival was the decrease of neutrophils (**C**,**D**) (∆Neutrophils < 0 median OS of 48.2 months vs. ∆Neutrophils ≥ 0 median OS of 20.5 months, *p*-value 0.008).

In our series, we found that the baseline expression of NLR, PCR, ESR and PCT above the median value was strongly correlated with a worse prognosis in the younger cohort of patients in terms of OS (Figure 2a,c,e and Figure 3a). On the other hand, no correlation could be demonstrated in the older cohort of patients (Figure 2b,d,f and Figure 3b). 

In the dynamic evaluation of ∆Parameters, only the treatment-related increase in neutrophil counts, evaluated as ΔN, correlated with a worse outcome in older patients with no effect in the younger cohort (Figure 3c,d).

#### 3.1.3. Prognostic Relevance of irAEs in Patients Treated with PD-1-Blocking mAbs

In the present study, we confirmed this correlation on the overall population (OS in patients with irAEs vs. those without irAEs: 20.5 (14.1–26.9) vs. 12.4 (7.4–17.4) *p* = 0.04) that showed similar features within the two age-related cohorts of patients but failed to achieve a statistical significance due to the low sample size (OS in younger patients with irAEs vs. without irAEs; 20.4 (17.8–23.0) vs. 11.4 (5.9–16.9) *p* = 0.13 and OS in older patients (≥ 75 years old) with irAEs vs. without irAEs; 25.0 (11.5–38.5) vs. 14.1 (3.0–25.1) *p* = 0.10).

## 4. Discussion

Our retrospective multi-institutional analysis conducted in a real-world setting confirmed the efficacy and safety of the PD-1-ICB treatment in patients older than 75 years with mNSCLC. No differences were observed in terms of PFS, OS and frequency of irAEs when compared with a cohort of younger patients (≤74 years old) who received a parallel treatment in the same time period. These data are in line with the results of previous studies reporting the efficacy and the safety of anti-PD-1 and anti-PD-L1 mABs in elderly patients [20,21,22,23]. The majority of these studies, however, chose a cut-off of 65 years according to the international guidelines and may not clearly reflect the actual elderly population affected by lung cancer. Due to the small number of elderly patients (≥75 years) with mNSCLC included in the clinical trials of immunotherapy and the issues of immunosenescence, the efficacy of PD-1-ICB among the subset of elderly patients remains unclear [13,24,25]. We chose the cut-off of 75 years as patients ≥ 75 years of age demonstrated the greatest disparity between the cancer diagnosis and the clinical trial representation [26]. It is noteworthy to underline that this cut-off was also chosen in many recent studies focusing on the efficacy of PD-1-ICB in elderly NSCLC [27,28]. The percentage of lung cancer cases reached 24.7% in patients ≥ 75 years [29] so our cohort was in line with the existing literature.

The novelty of our study is the investigation of the correlation between inflammation and the outcomes in age-dependent subsets of patients. We firstly evaluated if the patient outcome might be affected by a high inflammatory status at the treatment baseline and we found that high serum levels of most recognized inflammatory markers such as NLR, CRP, ESR and PCT were inversely correlated with the overall survival in the subset of younger patients. Conversely, these inflammatory markers did not correlate with survival in elderly patients. There is much evidence that a long-lasting inflammatory status in cancer patients may affect the existing balance between immune cell-mediated destruction and the growth of cancer cells in tumors, resulting in an accelerated progression of the disease and a worse prognosis [1,30,31,32,33,34]. We hypothesized, therefore, that elderly patients often present a much longer history of smoking and broncho-pneumopathies and, consequently, they achieve a different immunological habitus that is no longer affected by inflammation-related inhibitory pathways [20,21,22]. At the same time, the gut microbiome also plays a pivotal role in shaping systemic immune responses and is known to influence the efficacy of immune checkpoint inhibitors [35,36]. Despite the knowledge of the influence of sex, age and diet on the gut microbiome, little is known to what extent these parameters can affect the response to immunotherapy [37,38]. 

On the basis of these data, it can be hypothesized that the immune system and the inflammatory profile can act in different ways in younger and elderly subsets of patients. 

In elderly patients, a coexisting chronic inflammation in mNSCLC patients may still affect the immune balance between the immune system and cancer growth, affecting the anti-tumor activity of T cells and promoting the mechanisms of cancer-immune escape [39,40,41,42,43]. This hypothesis was supported by the rise in the neutrophil counts occurring upon ICB administration, predicting a worse prognosis in the older cohort of patients but not in the younger one where there was only a trend towards a significance. 

The correlation between the rise in the neutrophil counts and the poor outcomes could also be related to a rise in procalcitonin due to a bacterial influence or to other causes, as previously reported by our group [41].

In this regard, several studies have investigated different parameters and nomograms based on inflammation and various hematological variables in order to predict the prognosis of NSCLC patients such as the neutrophil/lymphocyte ratio [44], the thrombocyte/lymphocyte ratio [45], the C-reaction protein-to-albumin ratio [46] and the systemic immune inflammation index [47]. Despite the known effects of age on the inflammation profile [48,49], it is noteworthy to underline the insufficient exploration of this correlation in lung cancer. 

Palomar-Abril et al. recently investigated this correlation in a cohort of 124 patients (84 young and 40 older) and found a higher median CRP, a lower hemoglobin level and leukocyte count and a lower prognostic nutritional index in the older cohort [50]. Unfortunately, the authors did not correlate these parameters with the outcomes.

The investigation of the role of inflammation in lung cancer is pivotal to increase the therapeutic ratio especially in patients undergoing immunotherapy [51,52]. In this context, the relationship between the cancer cells and the immune system can be categorized into three cancer-immune phenotypes: the immune-desert phenotype, the immune-excluded phenotype and the inflamed phenotype [53]. Each phenotype is associated with different underlying biological mechanisms that aid the cancer cells to prevent the immune response of the host but the role of ageing of both the immune system and the patient should be better investigated to understand to what extent ageing can affect the outcomes of immunotherapy. Unfortunately, we were not able to perform this type of analysis in our retrospective evaluation. 

Finally, in previous studies, it has been shown that both the occurrence of irAEs and a rise in AAbs are strictly associated with the T cell engagement and/or rescue operated by an immunomodulating treatment including a PD-1 blockade [7]. It was shown that the occurrence of irAEs was often correlated with either the treatment response or a prolonged survival in several clinical studies including mNSCLC and colorectal cancer [7,54,55,56,57,58]. Our results are in line with the literature in both subsets of patients although the results failed to achieve a statistical significance due to the low sample size.

## 5. Conclusions

We can conclude that the age of mNSCLC patients with no severe comorbidities and a good performance status should not be considered as a criterium for excluding them from treatment with PD-1/PD-L1 mAbs. Additionally, on the basis of our results, we believe that a major effort should be made to understand and overcome the detrimental effect of systemic inflammation in mNSCLC patients in order to improve PD-1/PD-L1 blockade immunotherapy.

## Figures and Tables

**Table 1:** Distribution of the parameters (at the baseline and their variations during the immunotherapy: ∆Parameters) among the two cohorts of patients. A chi-squared analysis was used to compare the parameters among the two cohorts. Values are mean ± SD.

Parameters	Younger Patient Cohort (Age ≤ 74)	Older Patient Cohort (Age ≥ 75)	*p*-Values
Neutrophils	5.3 ± 3.5	4.6 ± 2.3	0.59
Lymphocytes	2.9 ± 10.4	1.4 ± 0.7	0.23
NLR	3.8 ± 3.7	3.5 ± 2.3	0.39
CRP	26.9 ± 42.7	18.8 ± 30.7	0.52
ESR	47.2 ± 31.8	49.7 ± 33.4	0.29
LDH	402.8 ± 286.7	373.3 ± 227.1	0.24
PCT	0.22 ± 0.50	0.1 ± 0.1	0.86
∆Neutrophils	7.5 ± 20.1	4.5 ± 9.1	0.18
∆Lymphocytes	1.5 ± 4.9	2.7 ± 4.6	0.23
∆NLR	8.5 ± 14.3	5.5 ± 7.2	0.06
∆CRP	10.2 ± 20.6	25.6 ± 71.7	0.14
∆ESR	8.8 ± 20.4	8.8 ± 21.9	0.14
∆LDH	2.7 ± 5.3	1.8 ± 5.1	0.70
∆PCT	7.3 ± 12.2	5.4 ± 6.9	0.54

## Data Availability

The data used to support the findings of this study are available from the corresponding author upon reasonable request.

## References

[1] Xia L., Liu Y., Wang Y. (2019). PD-1/PD-L1 Blockade Therapy in Advanced Non-Small-Cell Lung Cancer: Current Status and Future Directions. Oncologist.

[2] Feng M., Xiong G., Cao Z., Yang G., Zheng S., Song X., You L., Zheng L., Zhang T., Zhao Y. (2017). PD-1/PD-L1 and immunotherapy for pancreatic cancer. Cancer Lett..

[3] Friedman C.F., Proverbs-Singh T.A., Postow M.A. (2016). Treatment of the Immune-Related Adverse Effects of Immune Checkpoint Inhibitors: A Review. JAMA Oncol..

[4] Day D., Hansen A.R. (2016). Immune-Related Adverse Events Associated with Immune Checkpoint Inhibitors. BioDrugs.

[5] Naidoo J., Wang X., Woo K.M., Iyriboz T., Halpenny D., Cunningham J., Chaft J.E., Segal N.H., Callahan M.K., Lesokhin A.M. (2017). Pneumonitis in Patients Treated With Anti-Programmed Death-1/Programmed Death Ligand 1 Therapy. J. Clin. Oncol. Off. J. Am. Soc. Clin. Oncol..

[6] Giannicola R., D’Arrigo G., Botta C., Agostino R., Del Medico P., Falzea A.C., Barbieri V., Staropoli N., Del Giudice T., Pastina P. (2019). Early blood rise in auto-antibodies to nuclear and smooth muscle antigens is predictive of prolonged survival and autoimmunity in metastatic-non-small cell lung cancer patients treated with PD-1 immune-check point blockade by nivolumab. Mol. Clin. Oncol..

[7] Correale P., Saladino R.E., Giannarelli D., Sergi A., Mazzei M.A., Bianco G., Giannicola R., Iuliano E., Forte I.M., Calandruccio N.D. (2020). HLA Expression Correlates to the Risk of Immune Checkpoint Inhibitor-Induced Pneumonitis. Cells.

[8] Fulop T., Kotb R., Fortin C.F., Pawelec G., De Angelis F., Larbi A. (2010). Potential role of immunosenescence in cancer development. Ann. N. Y. Acad. Sci..

[9] Cusi M.G., Martorelli B., Di Genova G., Terrosi C., Campoccia G., Correale P. (2010). Age related changes in T cell mediated immune response and effector memory to Respiratory Syncytial Virus (RSV) in healthy subjects. Immun. Ageing.

[10] Hepper H.J., Sieber C., Walger P., Bahrmann P., Singler K. (2013). Infections in the elderly. Crit. Care Clin..

[11] Mutti L., Pentimalli F., Baglio G., Maiorano P., Saladino R.E., Correale P., Giordano A. (2020). Coronavirus Disease (Covid-19): What Are We Learning in a Country With High Mortality Rate?. Front. Immunol..

[12] Ferrara R., Mezquita L., Auclin E., Chaput N., Besse B. (2017). Immunosenescence and immunecheckpoint inhibitors in non-small cell lung cancer patients: Does age really matter?. Cancer Treat. Rev..

[13] Nosaki K., Saka H., Hosomi Y., Baas P., de Castro G., Reck M., Wu Y.-L., Brahmer J.R., Felip E., Sawada T. (2019). Safety and efficacy of pembrolizumab monotherapy in elderly patients with PD-L1–positive advanced non–small-cell lung cancer: Pooled analysis from the KEYNOTE-010, KEYNOTE-024, and KEYNOTE-042 studies. Lung Cancer.

[14] Correale P., Saladino R.E., Giannarelli D., Giannicola R., Agostino R., Staropoli N., Strangio A., Del Giudice T., Nardone V., Altomonte M. (2020). Distinctive germline expression of class I human leukocyte antigen (HLA) alleles and DRB1 heterozygosis predict the outcome of patients with non-small cell lung cancer receiving PD-1/PD-L1 immune checkpoint blockade. J. Immunother. Cancer.

[15] Seymour L., Bogaerts J., Perrone A., Ford R., Schwartz L.H., Mandrekar S., Lin N.U., Litière S., Dancey J., Chen A. (2017). iRECIST: Guidelines for response criteria for use in trials testing immunotherapeutics. Lancet Oncol..

[16] Cusi M.G., Botta C., Pastina P., Rossetti M.G., Dreassi E., Guidelli G.M., Fioravanti A., Martino E.C., Gandolfo C., Pagliuchi M. (2015). Phase I trial of thymidylate synthase poly-epitope peptide (TSPP) vaccine in advanced cancer patients. Cancer Immunol. Immunother..

[17] Correale P., Botta C., Martino E.C., Ulivieri C., Battaglia G., Carfagno T., Rossetti M.G., Fioravanti A., Guidelli G.M., Cheleschi S. (2015). Phase Ib study of poly-epitope peptide vaccination to thymidylate synthase (TSPP) and GOLFIG chemo-immunotherapy for treatment of metastatic colorectal cancer patients. OncoImmunology.

[18] Pastina P., Nardone V., Botta C., Croci S., Tini P., Battaglia G., Ricci V., Cusi M.G., Gandolfo C., Misso G. (2017). Radiotherapy prolongs the survival of advanced non-small-cell lung cancer patients undergone to an immune-modulating treatment with dose-fractioned cisplatin and metronomic etoposide and bevacizumab (mPEBev). Oncotarget.

[19] Martino E.C., Misso G., Pastina P., Costantini S., Vanni F., Gandolfo C., Botta C., Capone F., Lombardi A., Pirtoli L. (2016). Immune-modulating effects of bevacizumab in metastatic non-small-cell lung cancer patients. Cell Death Discov..

[20] Elias R., Morales J., Presley C. (2017). Checkpoint Inhibitors for Non-Small Cell Lung Cancer Among Older Adults. Curr. Oncol. Rep..

[21] Muchnik E., Loh K.P., Strawderman M., Magnuson A., Mohile S.G., Estrah V., Maggiore R.J. (2019). Immune Checkpoint Inhibitors in Real-World Treatment of Older Adults with Non–Small Cell Lung Cancer. J. Am. Geriatr. Soc..

[22] Youn B., Trikalinos N.A., Mor V., Wilson I.B., Dahabreh I.J. (2019). Real-world use and survival outcomes of immune checkpoint inhibitors in older adults with non–small cell lung cancer. Cancer.

[23] Tini P., Nardone V., Pastina P., Pirtoli L., Correale P., Giordano A. (2018). The effects of radiotherapy on the survival of patients with unresectable non-small cell lung cancer. Expert Rev. Anticancer. Ther..

[24] Yamaguchi O., Imai H., Minemura H., Suzuki K., Wasamoto S., Umeda Y., Osaki T., Kasahara N., Uchino J., Sugiyama T. (2020). Efficacy and safety of immune checkpoint inhibitor monotherapy in pretreated elderly patients with non-small cell lung cancer. Cancer Chemother. Pharmacol..

[25] Nikolich-Žugich J. (2018). The twilight of immunity: Emerging concepts in aging of the immune system. Nat. Immunol..

[26] Pang H., Wang X., Stinchcombe T.E., Wong M.L., Cheng K.M., Ganti A.K., Sargent D.J., Zhang Y., Hu C., Mandrekar S.J. (2016). Enrollment Trends and Disparity Among Patients With Lung Cancer in National Clinical Trials, 1990 to 2012. J. Clin. Oncol..

[27] Takamori S., Shimokawa M., Komiya T. (2021). Prognostic impact of chronological age on efficacy of immune checkpoint inhibitors in non-small-cell lung cancer: Real-world data from 86 173 patients. Thorac. Cancer.

[28] Morimoto K., Yamada T., Yokoi T., Kijima T., Goto Y., Nakao A., Hibino M., Takeda T., Yamaguchi H., Takumi C. (2021). Clinical impact of pembrolizumab combined with chemotherapy in elderly patients with advanced non-small-cell lung cancer. Lung Cancer.

[29] Sung H., Ferlay J., Siegel R.L., Laversanne M., Soerjomataram I., Jemal A., Bray F. (2021). Global Cancer Statistics 2020: GLOBOCAN Estimates of Incidence and Mortality Worldwide for 36 Cancers in 185 Countries. CA Cancer J. Clin..

[30] Thompson J.C., Hwang W.-T., Davis C., Deshpande C., Jeffries S., Rajpurohit Y., Krishna V., Smirnov A., Verona R., Lorenzi M.V. (2020). Gene signatures of tumor inflammation and epithelial-to-mesenchymal transition (EMT) predict responses to immune checkpoint blockade in lung cancer with high accuracy. Lung Cancer.

[31] Danaher P., Warren S., Lu R., Samayoa J., Sullivan A., Pekker I., Wallden B., Marincola F.M., Cesano A. (2018). Pan-cancer adaptive immune resistance as defined by the Tumor Inflammation Signature (TIS): Results from The Cancer Genome Atlas (TCGA). J. Immunother. Cancer.

[32] Liu C., Zheng S., Jin R., Wang X., Wang F., Zang R., Xu H., Lu Z., Huang J., Lei Y. (2020). The superior efficacy of anti-PD-1/PD-L1 immunotherapy in KRAS-mutant non-small cell lung cancer that correlates with an inflammatory phenotype and increased immunogenicity. Cancer Lett..

[33] Bar N., Costa F., Das R., Duffy A., Samur M., McCachren S., Gettinger S.N., Neparidze N., Parker T.L., Bailur J.K. (2020). Differential effects of PD-L1 versus PD-1 blockade on myeloid inflammation in human cancer. JCI Insight.

[34] Correale P., Botta C., Staropoli N., Nardone V., Pastina P., Ulivieri C., Gandolfo C., Baldari T.C., Lazzi S., Ciliberto D. (2018). Systemic inflammatory status predict the outcome of k-RAS WT metastatic colorectal cancer patients receiving the thymidylate synthase poly-epitope-peptide anticancer vaccine. Oncotarget.

[35] Hakozaki T., Richard C., Elkrief A., Hosomi Y., Benlaïfaoui M., Mimpen I., Terrisse S., DeRosa L., Zitvogel L., Routy B. (2020). The Gut Microbiome Associates with Immune Checkpoint Inhibition Outcomes in Patients with Advanced Non–Small Cell Lung Cancer. Cancer Immunol. Res..

[36] Shaikh F.Y., Gills J.J., Sears C.L. (2019). Impact of the microbiome on checkpoint inhibitor treatment in patients with non-small cell lung cancer and melanoma. EBioMedicine.

[37] Vogl T., Klompus S., Leviatan S., Kalka I.N., Weinberger A., Wijmenga C., Fu J., Zhernakova A., Weersma R.K., Segal E. (2021). Population-wide diversity and stability of serum antibody epitope repertoires against human microbiota. Nat. Med..

[38] Cui M., Trimigno A., Aru V., Rasmussen M.A., Khakimov B., Engelsen S.B. (2021). Influence of Age, Sex, and Diet on the Human Fecal Metabolome Investigated by 1H NMR Spectroscopy. J. Proteome Res..

[39] Garner H., De Visser K.E. (2020). Immune crosstalk in cancer progression and metastatic spread: A complex conversation. Nat. Rev. Immunol..

[40] Jiang X., Wang J., Deng X., Xiong F., Ge J., Xiang B., Wu X., Ma J., Zhou M., Li X. (2019). Role of the tumor microenvironment in PD-L1/PD-1-mediated tumor immune escape. Mol. Cancer.

[41] Nardone V., Giannicola R., Bianco G., Giannarelli D., Tini P., Pastina P., Falzea A.C., Macheda S., Caraglia M., Luce A. (2021). Inflammatory Markers and Procalcitonin Predict the Outcome of Metastatic Non-Small-Cell-Lung-Cancer Patients Receiving PD-1/PD-L1 Immune-Checkpoint Blockade. Front. Oncol..

[42] Nardone V., Pastina P., Giannicola R., Agostino R., Croci S., Tini P., Pirtoli L., Giordano A., Tagliaferri P., Correale P. (2018). How to Increase the Efficacy of Immunotherapy in NSCLC and HNSCC: Role of Radiation Therapy, Chemotherapy, and Other Strategies. Front. Immunol..

[43] Correale P., Saladino R.E., Nardone V., Giannicola R., Agostino R., Pirtoli L., Caraglia M., Botta C., Tagliaferri P. (2019). Could PD-1/PDL1 immune checkpoints be linked to HLA signature?. Immunotherapy.

[44] Grieshober L., Graw S., Barnett M.J., Goodman G.E., Chen C., Koestler D.C., Marsit C.J., Doherty J.A. (2021). Pre-diagnosis neutrophil-to-lymphocyte ratio and mortality in individuals who develop lung cancer. Cancer Causes Control..

[45] Seber S., Bayir D., Yetisyigit T. (2020). Prognostic values of various hematological variables as markers of systemic inflammation in metastatic lung cancer. J. Cancer Res. Ther..

[46] Huang Z., Xing S., Zhu Y., Qu Y., Jiang L., Sheng J., Wang Q., Xu S., Xue N. (2020). Establishment and Validation of Nomogram Model Integrated With Inflammation-Based Factors for the Prognosis of Advanced Non-Small Cell Lung Cancer. Technol. Cancer Res. Treat..

[47] Biswas T., Kang K., Gawdi R., Bajor D., Machtay M., Jindal C., Efird J. (2020). Using the Systemic Immune-Inflammation Index (SII) as a Mid-Treatment Marker for Survival among Patients with Stage-III Locally Advanced Non-Small Cell Lung Cancer (NSCLC). Int. J. Environ. Res. Public Heal..

[48] Davizon-Castillo P., McMahon B., Aguila S., Bark D., Ashworth K., Allawzi A., Campbell R.A., Montenont E., Nemkov T., D’Alessandro A. (2019). TNF-α–driven inflammation and mitochondrial dysfunction define the platelet hyperreactivity of aging. Blood.

[49] Bergholt N.L., Olesen M.L., Foldager C.B. (2019). Age-Dependent Systemic Effects of a Systemic Intermittent Hypoxic Therapy In Vivo. High Alt. Med. Biol..

[50] Palomar-Abril V., Comes T.S., Campos S.T., Ureste M.M., Bosch V.G., Maiques I.C.M. (2020). Impact of Age on Inflammation-Based Scores among Patients Diagnosed with Stage III Non-Small Cell Lung Cancer. Oncology.

[51] Vareki S.M. (2018). High and low mutational burden tumors versus immunologically hot and cold tumors and response to immune checkpoint inhibitors. J. Immunother. Cancer.

[52] Bonaventura P., Shekarian T., Alcazer V., Valladeau-Guilemond J., Valsesia-Wittmann S., Amigorena S., Caux C., Depil S. (2019). Cold Tumors: A Therapeutic Challenge for Immunotherapy. Front. Immunol..

[53] Chen D.S., Mellman I. (2017). Elements of cancer immunity and the cancer-immune set point. Nature.

[54] Läubli H., Koelzer V.H., Matter M.S., Herzig P., Schlienger B.D., Wiese M.N., Lardinois D., Mertz K., Zippelius A. (2017). The T cell repertoire in tumors overlaps with pulmonary inflammatory lesions in patients treated with checkpoint inhibitors. OncoImmunology.

[55] Fiala O., Sorejs O., Sustr J., Kucera R., Topolcan O., Finek J. (2020). Immune-related Adverse Effects and Outcome of Patients With Cancer Treated With Immune Checkpoint Inhibitors. Anticancer. Res..

[56] Correale P., Tagliaferri P., Fioravanti A., Del Vecchio M.T., Remondo C., Montagnani F., Rotundo M.S., Ginanneschi C., Martellucci I., Francini E. (2008). Immunity Feedback and Clinical Outcome in Colon Cancer Patients Undergoing Chemoimmunotherapy with Gemcitabine + FOLFOX followed by Subcutaneous Granulocyte Macrophage Colony-Stimulating Factor and Aldesleukin (GOLFIG-1 Trial). Clin. Cancer Res..

[57] Correale P., Fioravanti A., Bertoldi I., Montagnani F., Miracco C., Francini G. (2008). Occurrence of Autoimmunity in a Long-Term Survivor with Metastatic Colon Carcinoma Treated with a New Chemo-Immunotherapy Regimen. J. Chemother..

[58] Nardone V., Tini P., Pastina P., Botta C., Reginelli A., Carbone S., Giannicola R., Calabrese G., Tebala C., Guida C. (2019). Radiomics predicts survival of patients with advanced non-small cell lung cancer undergoing PD-1 blockade using Nivolumab. Oncol. Lett..

